# PILOT STUDY OF COMBINED TRANSVERTEBRAL MAGNETIC AND TRANSCUTANEOUS STIMULATION FOR THE REHABILITATION OF COMBAT ACUTE SPINAL CORD INJURIES

**DOI:** 10.2340/jrm-cc.v8.42686

**Published:** 2025-07-09

**Authors:** Oleksandr Kulyk, Ivan Mazurchuk, Valeriia Polousova, Anna Pshenychna, Oksana Yarmolenko

**Affiliations:** 1 Department Research Neurological and Neurosurgical Rehabilitation, Neurological and Neurosurgical Rehabilitation Research Centre “NODUS”, Brovary, Kyiv region, Ukraine; 2 Department Physiotherapy, Neurological and Neurosurgical Rehabilitation Research Centre “NODUS”, Brovary, Kyiv region, Ukraine; 3 Department Ergotherapy, Neurological and Neurosurgical Rehabilitation Research Centre “NODUS”, Brovary, Kyiv region, Ukraine; 4 Department Assistive Technology, Neurological and Neurosurgical Rehabilitation Research Centre “NODUS”, Brovary, Kyiv region, Ukraine; 5 Department Neurophysiology, Neurological and Neurosurgical Rehabilitation Research Centre “NODUS”, Brovary, Kyiv region, Ukraine

**Keywords:** transvertebral magnetic stimulation for spinal cord injury, non-invasive electrical stimulation of peripheral nerves, early neurorehabilitation, combating spinal cord injury

## Abstract

**Aim of the study:**

To improve the effectiveness of neurorehabilitation in patients with severe combat spinal cord injury by combining spinal cord repetitive transvertebral magnetic stimulation (rTvMS) and non-invasive transcutaneous electrical stimulation (TcES) of peripheral nerves.

**Clinical rationale for study:**

For the best recovery from severe combat spinal cord injury, neurorehabilitation must start in the acute phase. Only technologies targeting sensorimotor conduction and functional improvement can confirm the potential of the time factor. Non-invasive neuromodulation has been shown to work for combat spinal cord injury of varying severity.

**Material and methods:**

We have analysed 154 cases of severe combat spinal cord injury, followed continuously for at least 12 months from the start of neurorehabilitation. A unified «end-to-end» protocol combined rTvMS of the spinal cord with simultaneous TcES of peripheral nerves in different modes was developed for non-invasive spinal cord neuromodulation.

**Results:**

The combination of these parameters produced the most positive results in post-traumatic sensory-motor disorders: (*i*). rTvMS, level ThX-LI: 2000 pulses per set, 100 pulse packages, 5–10 Hz, intensity “+ 30––40%” of the threshold of the evoked motor potential; TcES n. tibialis or n. peroneus: 5–10 Hz, pulse intensity corresponded to the threshold of the motor response, functional electrical stimulation (FES) mode. (*ii*). rTvMS, level C_II_-Th_II_: 2000 pulses per set, 50 pulse packages, 5–7 Hz, intensity + 20–30% of the threshold of the evoked motor potential; TcES n. medianus or n. ulnaris; n. tibialis or n. peroneus: 5–10 Hz, pulse intensity corresponded to the threshold of the motor response, FES mode. Approximately 28% of patients in group A (FRANKEL/ASIA) moved to a higher level of function after 3 courses of neurorehabilitation intervention (90 working days).

**Conclusions and clinical implications:**

Electro-magnetic stimulation of the spinal cord excitatory cell conduction system according to the principle of “end-to-end: as in Hebb’s theory,” combined with physical movement, led to an increase in spinal cord conduction in the acute phase of combat spinal cord injury. This was manifested by neurological and functional improvement.

## LAY ABSTRACT

If you imagine, for example, that the ability to move a person’s legs or arms depends on 2 nerve cells that are connected and form a kind of conductor or bridge, the ends of which are these cells, then any physical impact that breaks this contact will lead to the loss of this ability. Now, if we imagine that for every cell that has lost contact with another, we act in such a way that, under the influence of this action, they re-establish this connection; it is evident that this can lead to the restoration of movement. It is this «end-to-end» effect, that is simultaneously from both ends with electromagnetic waves of specific parameters on spinal cord cells above and below the level of damage, in combination with specific physical exercises, that led to the improvement of motor and sensory functions after severe gunshot spinal cord injury.

The Ukrainian medical system is faced with a significant influx of young and middle-aged patients requiring long-term neurorehabilitation after severe combat spinal cord injury (CSCI). Unlike civilian trauma, mine-explosive and gunshot injuries to the spine and spinal cord are clinically much more severe and have a worse prognosis for neurological recovery (1).

For the best recovery after CSCI, neurorehabilitation should be started in the acute phase (2). To make the most of the time factor, we need technologies that are based on evidence and target sensorimotor conduction and functional improvement (3, 4). Spinal conduction processes are very important in the current clinical challenges and in selecting approaches to comprehensive neurorehabilitation for all types and severities of CSCI (5, 6).

The rapid rise in disability and mortality rates in the most able-bodied and reproductive segments of the population is a major threat to Ukraine’s medical and demographic situation. The experience of Ukraine shows that aggressive militarisation can have similar medical and social consequences for every European country.

We initiated this study to improve the effectiveness of neurorehabilitation methods for critically ill patients using neuromodulation technologies and to analyse the used protocols to find the best solution among those used by us. This will increase their functional capacity and independence, reducing pressure on their families and promoting effective practices in similar situations.

## MATERIAL AND METHODS

### Design and participants

This study was conducted as a pilot observational prospective–retrospective cohort analysis with elements of a case‑control design. It included 154 cases of medical neurorehabilitation intervention for CSCI recorded between August 2014 and August 2024. The inclusion criteria were:

1. Combat (gunshot, mine-explosive) CSCI of various localisations.
2. Both sex and age from 18 to 60 years.
3. Single segment spine injury.
4. Absence of non-medical factors that would influence the volume and intensity of rehabilitation care throughout the observation period.
5. Start of neurorehabilitation in up to 1 month from the date of CSCI.
6. The total uninterrupted duration of neurorehabilitation courses is at least 12 months.
7. Duration of observation 12–36 months.
8. Negative or positive outcome of neurorehabilitation at the time of discharge from the rehabilitation hospital.

The exclusion criteria were:

1. Secondary spinal cord injuries due to the progression of infected gunshot wounds in the neck, chest or abdomen and the spread of swelling or inflammation to the spine and spinal cord.
2. The presence of burns above the third degree involving the area of gunshot damage to the spine and spinal cord
3. Persistent pain syndrome that was aggravated using magnetic or electrical stimulation technologies.
4. Violation of treatment compliance by the patient.

After applying predefined inclusion and exclusion criteria, 192 patients were considered for inclusion. Of these, 154 met the final criteria and were included in the analysis; their clinical datasets formed the empirical foundation of this study.

The clinical case follow-up and early neurorehabilitation started at the same time. Due to the special needs of combat injuries, neurorehabilitation could only begin after neurosurgical treatment. All events were counted from this starting point, not from the time of injury or other periods, as is common in the literature for civilian trauma.

Due to the different clinical conditions in the acute and post-acute phases of CSCI, rehabilitation measures may vary (7). In Ukraine, rehabilitation care in a hospital setting is considered high volume if it lasts 3 h per day or more (8). The volume of rehabilitation care is also determined by the capacity of a medical institution and its organisation (9, 10). In this study, the volume of 3-h rehabilitation activities was taken as the basic reference, to which all other hours of care were added. This determined the daily volume and intensity of care.

To create a statistical series of the ordered distribution of CSCI cases into groups according to the intensity of rehabilitation care, that is the number of hours per day or week, the volume of neurorehabilitation was classified as follows: low intensity for on average 3 or less additional hours per day (< 30 h per week in total). Four or more hours of work added to the basic 3 h per day (35–50 h per week) was considered high intensity. In some cases, (*n* = 3), rehabilitation care lasted 31–34 h, so it was divided according to the following principle: 31–32 h per week were classified as low intensity (*n* = 2) and 33–34 h as high intensity (*n* = 1).

Spinal cord functions were non-invasively stimulated at different frequencies (1–10 Hz) using magnetic impulses of rhythmic magnetic transvertebral stimulation (rTvMS) at 1 cm from the body surface. These sites are standardised for the study of «peripheral» motor evoked potentials. Hardware modes: monophasic, biphasic theta-burst stimulation (TBS), paired stimulation with magnetic induction amplitude up to 3 T and intensity up to 10,000 stimuli. We have used Ukrainian-made software with a State Certificate of Conformity: UA1.177.0064894-13. The ring coil of the magnetic stimulator was cooled.

Non-invasive percutaneous electrical stimulation (TcES) was used to stimulate peripheral nerves. A contact stimulation electrode was applied to standardised sites to determine nerve responses. Hardware modes: neuromuscular electrical stimulation (NMES), functional electrical stimulation (FES) [combined with hardware, robotic kinesitherapy (11)] were supplemented by digital dynamometry and neurophysiological parameters of motor or sensory impulse conduction on an electromyographic apparatus with 4 channels of electrical stimulation and software of Ukrainian (UA) and foreign (D) production. State certificate of conformity: UA1.177.0064894-13 TU U 33.1-30428373-004-2004 and CE006.01005.001.

To standardise non-invasive neuromodulation sessions, a local “end-to-end” clinical protocol was developed and approved (No: NMFS-58 «Differential use of non-invasive neuromodulation in combination with electrical stimulation of peripheral nerves in CSCI to improve motor and sensory conduction of nerve impulses»), which is published on the clinic website.

The concept underlying the development of this protocol (see Fig. 3) is based on Hebb’s theory – «Hebb’s learning postulate» (12–14).

Neuromodulation was applied in courses of 20 sets, performed daily in one or more (not more than 3) sessions. The average session lasted 45 min, with a 1-h break between them in case of several sessions.

All patients who met the inclusion criteria received a neurorehabilitation programme that incorporated neuromodulation technologies in accordance with the protocol described later.

Clinical status and neurological status were recorded according to the International Standards for Neurological Classification of Spinal Cord Injury (ISNCCSCI) (15). The degree of sensorimotor impairment and the level of preserved (partially preserved) function were assessed using the ASIA and FRANKEL scales (15–17) at the start of neurorehabilitation and after 3, 6 and 12 months.

We have used clinical guidelines to manage acute CSCI in our neurorehabilitation department (18, 19).

The parameters of robotic kinesitherapy sessions were dosed according to kinematic physical indicators: angular velocity, torque, pace, force, work and coefficient of variation for each passive/active physical movement. These aspects were monitored in real time, statistically processed and compared with clinical outcomes.

The functional tests (20) used in the study were as follows:

- 6-minute walk test (6MWT).
- 10-meter walk test (10MWT).
- Berg Balance Scale.
- Timed Up and Go Test (TUG).
- Graded redefined assessment of strength sensibility and prehension (GRASSP).
- Modified functional reach test (mFRT).
- Spinal cord independence measure, version III (CSCIM-III).

Non-invasive neuromodulation of the spinal cord has clinical effects that take time to manifest, due to the principle of “accumulation of learning stimulus” (12).

In our study, electromagnetic exposure was evaluated during the first 3 months.

A 30-day medical neurorehabilitation course (1 cycle) included individual neurorehabilitation plans consisting of the following components:

1. physical therapy sessions [robotic mechanotherapy with biofeedback, PNF-therapy (proprioceptive neuromuscular facilitation), ROM-therapy (range of motion exercises), CPM-therapy (continuous passive motion), verticalisation therapy, basic therapeutic exercise, balance and proprioceptive training, GT-therapy (gait training)].
2. sessions of non-invasive neuromodulation of spinal cord functions [repetitive transvertebral magnetic stimulation of the spinal cord (rTvMS) with simultaneous electrical stimulation of peripheral nerves (TcES)].
3. occupational therapy sessions (splinting and adaptive equipment, sensory integration activities, therapeutic exercises, environmental modifications, motor-eyes activities).
4. orthotics therapy.
5. bladder and digestive tract management.
6. psychotherapy sessions.

The data are completely depersonalised, and no experiments were performed on patients.

Medications used for symptomatic treatment during neurorehabilitation were used according to their marketing authorisation and instructions for use.

The decision to prescribe rehabilitation methods or medications was separate from the decision to include the patient in the trial.

Patients did not undergo any additional procedures for data analysis or verification or for statistical purposes.

Each patient gave written consent to medical rehabilitation intervention using approved local clinical protocols, was informed and aggraded that his or her data would be processed.

At the time of CSCI, all patients were considered fit for military service in wartime by the Military Medical Commission.

## RESULTS

The mean age of patients at the time of injury was 39 ± 6 years (range: 23–56). The overwhelming majority of participants were male (98.05%, *n* = 151), while only 1.95% (*n* = 3) were female.

All cases of CSCI were associated with concomitant damage to soft tissues and/or internal organs caused by ballistic or blast-related trauma. Although all patients sustained gunshot-induced vertebral fractures, not every case involved penetration of the spinal canal by either the traumatic agent or displaced bone fragments. In 43% of patients, the spinal canal was radiologically and intraoperatively intact. These injuries were attributed to high-energy impacts and brief compressive forces. In contrast, 57% of cases involved direct impact followed by prolonged compression, with retained foreign bodies identified. Notably, 24% (*n* = 21) of patients exhibited penetrating spinal cord injuries, characterised by partial transection or dissection of neural tissue. The distribution of clinical cases according to the level of damage to the spine and spinal cord is as follows:

- 12.5% (*n* = 19) – cervical spine (most frequently motor level C5–C6 – *n* = 10: relative frequency – W _C5–C6_ = 0.52).
- 19% (*n* = 29) – thoracic spine (most frequently motor level Th10–Th12 – *n* = 19: relative frequency – W _Th10–Th12_ = 0.65).
- 44% (*n* = 67) – lumbar spine (most frequently motor level L2–L3 – *n* = 39: relative frequency – W _L2–L3_ = 0.58).
- 24.5% (*n* = 39) – sacral spine (most frequently motor level S1–S2 – *n* = 21: relative frequency – W _S1–S2_ = 0.53).

The distribution pattern of combat-related mine-explosive and gunshot injuries to the spine and spinal cord demonstrated a statistically significant correlation with the anatomical positioning of individual body armour components (*r* > 0.71), as well as with their quality or absence (*r* > 0.79).

All 154 patients underwent primary neurosurgical intervention, including decompression, within 120 h of injury (range: 6–120 h). However, the mean time to surgical intervention was 36 ± 7 h, which notably exceeds the generally accepted threshold for early decompression of < 24 h (21, 22).

Nearly one-third of patients (*n* = 48) required a second surgical procedure – such as wound debridement, stabilisation or repeated decompression – within 72 h of the initial surgery. Importantly, no case demonstrated a measurable change in neurological status following reintervention.

**Fig. 1:**
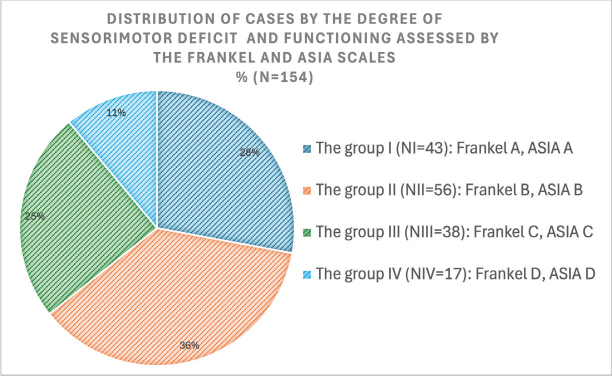
In this study, patients with sensorimotor deficit level B (incomplete) predominated at baseline. The total number of cases with incomplete CSCI was 72%.

All patients completed their neurosurgical treatment within 14 days of injury and were subsequently transferred to a specialised neurorehabilitation facility equipped with an intensive care unit. Neurosurgical supervision continued for an additional 2–3 weeks as part of a multidisciplinary care team. Notably, 62.33% (*n* = 96) of patients required ongoing intensive care during the early neurorehabilitation period. The distribution of the 154 cases according to the level of the input sensorimotor deficit and the functioning according to the scores and levels of the FRANKEL/ASIA scales at the beginning of the observation is shown in Fig. 1. Two similar scales were used during the study ASIA scale and the FRANKEL scale to determine the level of neurological deficit. This is also reflected in Scientific publications, where the FRANKEL scale is considered outdated by some (23) and actively used by others (24, 25).

Only 44.15% of patients (*n* = 154, *n* = 68) underwent high-intensity neurorehabilitation during the first month of observation (the total duration of classes and procedures in hours was on average 7 h per day and about 40 h per week). The condition of the remaining patients during the same period forced them to choose a low-intensity tactic (on average 4 h per day and about 20 h per week). A comparison of the schedules showed that main difference between the different intensities was the number of non-invasive neuromodulation sessions per day (respectively per week) in combination with hardware kinesitherapy (FES mode) or without it (NMES mode). This includes 3 sessions for high intensity and 1 session for low intensity neurorehabilitation care.

**Fig. 2:**
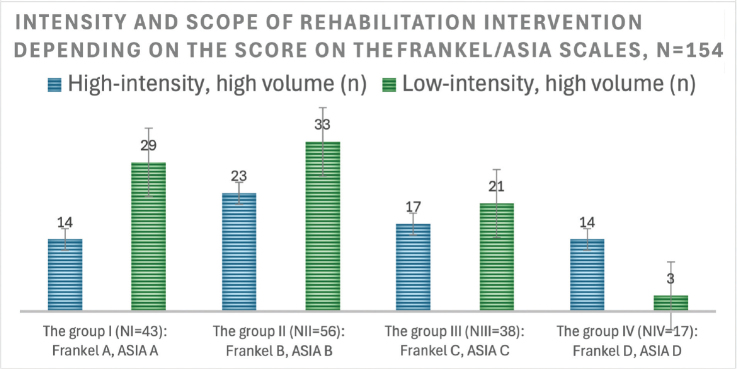
Low-intensity and high-volume neurorehabilitation activities dominated. An analysis shows that patients in these groups had psychological or neurological complaints that prevented them from attending 7–9 h of rehabilitation sessions in the first 3 months. Some complained of not being able to withstand the load, some of the intensity of the classes, some of frequent wound dressings, some of contact ulcers and long-term back pain and some of external fixation devices for gunshot fractures.

The distribution of the volume and intensity of neurorehabilitation as a function of the scores on the FRANKEL/ASIA scales in the first month of observation is shown in Fig. 2.

**Fig. 3:**
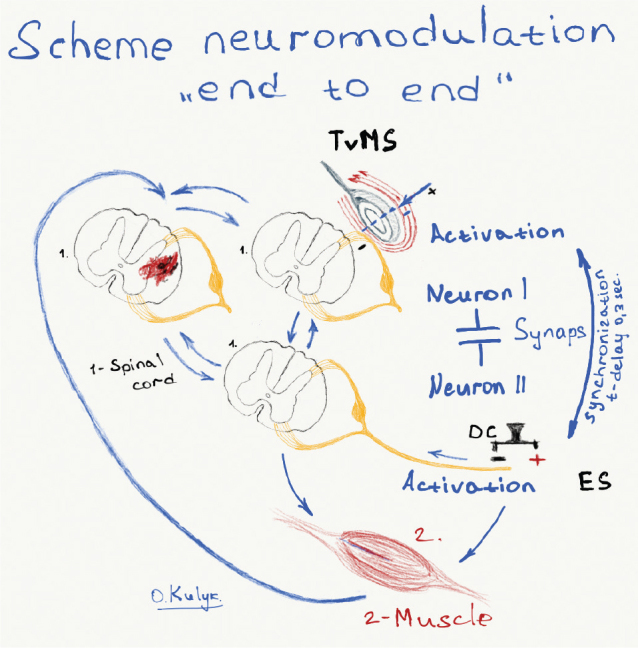
The development of this protocol is based on Hebb’s postulate of associative learning at the cellular level between 2 neurons, leading to a constant modification of the activity pattern of a spatially distributed ensemble of nerve cells. The learning process means that new connections are formed, and existing connections are strengthened (the strength of the connection increases) between 2 mutually active neurons involved in constant or periodic activation of each other over time (12, 13). TVMS: transvertebral magnetic stimulation; ES: electrical stimulation. The idea behind this protocol is as follows. They are assuming that during a spinal cord injury, there are conditions where the preserved connections between neurons have a weak strength, or there are opportunities for 2 neurons to form new connections when activating each other, increasing their strength over time on a microscale, which has no clinical manifestation on a macroscale. A cellular network with multidirectional impulse movement can be hypothetically and simplistically considered a conductor with 2 ends, where excitatory elements are at the ends, and in the middle, there is a functional «weak» synapse or a damaged «synapse» or a «traumatic obstacle» that disrupts the impulse. Thus, there are substantial grounds for the fact that systematic, prolonged almost synchronous (with a certain delay) electromagnetic stimulation of a conductor from both ends (above and below the level of the obstacle or weak synapse) can create conditions for strengthening the “weak synapse” or adaptive bypass of the functional obstacle through a new connection between the 2 ends of the conductor, that is neurons, which will already have a positive effect on the macro scale. The “end-to-end” protocol was developed for such synchronous (simultaneous) stimulation, using transvertebral magnetic stimulation of the spinal cord and electrical stimulation with direct current of the peripheral nerves and indirectly of the spinal cord. In the figure, the image of the coil means that the magnetic effect is applied above the location of the “functional obstacle or ‘weak’ synapse” and the simultaneous effect with a specific short time delay of direct electric current on the nerve area below the level of the functional obstacle in the NMES mode and with motor muscle reinforcement in the FES mode.

**Fig. 4:**
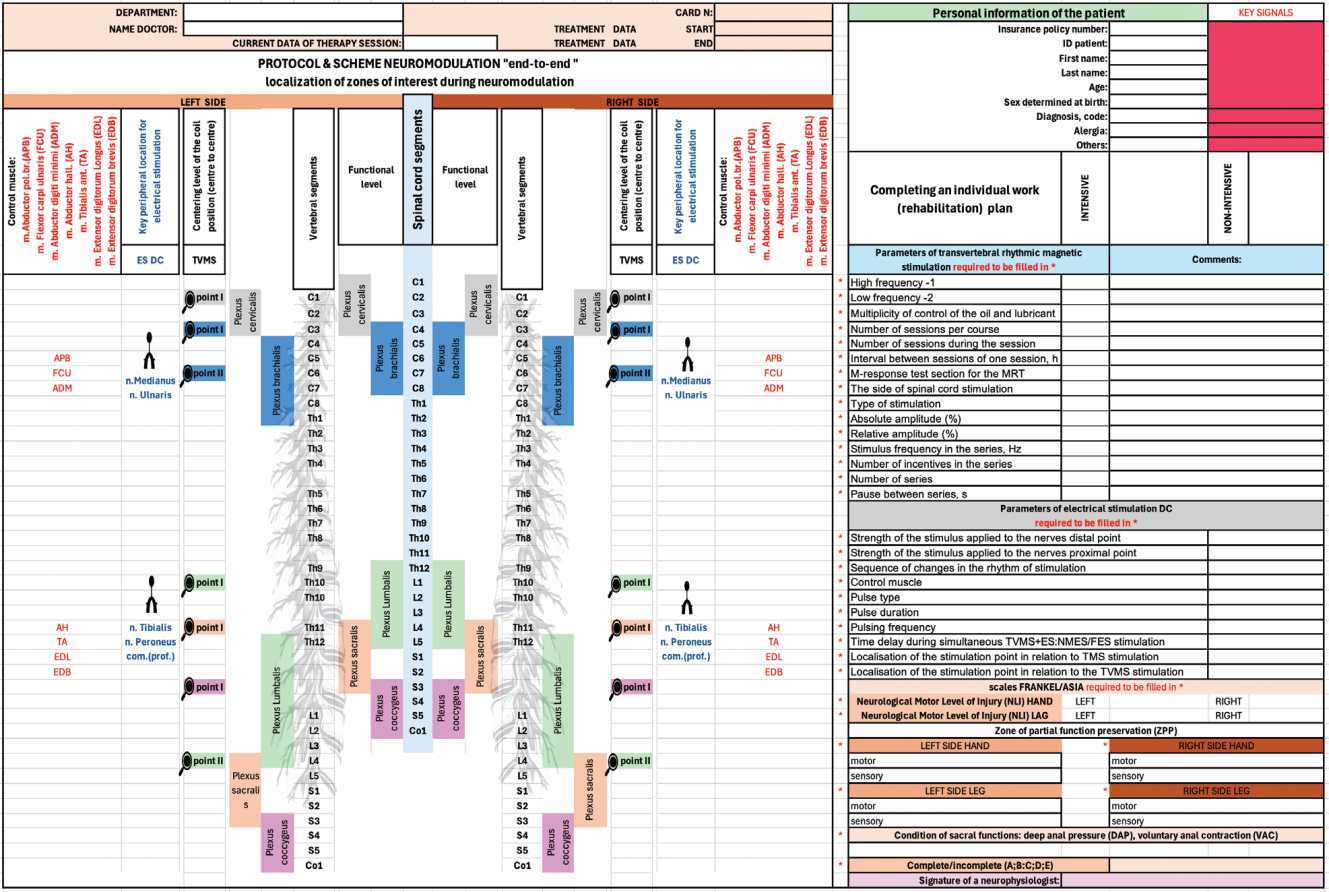
Depending on the topographic level of spinal cord damage according to the defined FRANKEL/ASIA zone of partially preserved function, select the appropriate projection locations for transvertebral spinal cord stimulation and simultaneous stimulation of the corresponding functional peripheral nerves on the same side of the stimulation and set the appropriate time delay in seconds to determine the sequence of stimuli (1 stimulus should be preceding). The functional obstacle should be hypothetically located between the home ends of an imaginary conductor to which stimuli are applied. Choose different combinations: TMVS+ES (NMES or FES).

The conceptual flowchart of the “end-to-end” protocol and the protocol itself is shown in Figs. 3 and 4, respectively.

Tables I and II show the summary parameters of neuromodulation according to the “end-to-end” protocol used in this study. Of all the variants of neuromodulation settings, the following combination contributed most to the reproducible positive dynamics of post-traumatic sensorimotor dysfunction in the acute and post-acute phases of CSCI: (*i*) rTvMS, level Th_X_-L_I_: 2000 pulses per set, 100-pulse bursts, frequency 5–10 Hz, intensity “+ 30–40%” of the threshold of the evoked motor potential; TcES n. tibialis or n. peroneus: frequency 5–10 Hz, pulse intensity corresponding to the threshold of the motor response, FES mode. (*ii*) rTvMS, level C_II_-Th_II_: 2000 pulses per set, 50-pulse bursts, frequency 5–7 Hz, intensity + 20–30% of the threshold of the evoked motor potential; TcES n. medianus or n. ulnaris; n. tibialis or n. peroneus: frequency 5–10 Hz, pulse intensity corresponding to the threshold of the motor response, FES mode.

**Table I:** Summary protocols of rhythmic TVMS

Clinical task	Motor and sensory improvement – hands		Motor and sensory improvement – legs	
Stimulation mode				
High frequency -1	1	2	1	2
Low frequency -2				
Multiplicity of control of the oil and lubricant	Every 5 sessions	Every 10 sessions	Every 5 sessions	Every 5 sessions
Number of sessions per course	20	20	20	20
Number of sessions during the session	3	3	3	3
The interval between sessions of 1 session, h	3	5	5	8
M-response test section for the MRT	APB	APB	AH	AH
The side of spinal cord stimulation	Left, right	Left, right	Left, right	Left, right
Type of stimulation	Biphasic	Biphasic	Biphasic	Biphasic
Absolute amplitude (%)	70–100	100	70–100	100
Relative amplitude (%)	70–110	110–130	70–80	80–100
Stimulus frequency in the series, Hz	5; 10; 20	0.5; 1	10; 20	0.25; 0.5; 1
Number of incentives in the series	50	50	100	100
Number of series	20	20	20	20
Pause between series s	5; 10; 20	1; 5	20	20

APB: m. abductor policis brevis, AH: m. abductor hallucis; MRT: motor response threshold; TVMS: transvertebral magnetic stimulation

**Table II:** Summary protocols of electrical nerve stimulation (ENS)

Strength of the stimulus applied to the n. medianus or n. tibialis at the distal point	Equal to the threshold peripheral motor response
Strength of the stimulus applied to the n. medianus or n. tibialis at the distal point	After 3 min, the current strength was increased by 20% of the motor response threshold, reaching the second (neural) electrostimulation horizon. Visual muscle contractions confirmed this, creating a dynamogenic effect.
The sequence of changes in the rhythm of stimulation	A continuous rhythm at a frequency of 70–120 Hz > fast > slow > slow > fairly fast (15–20% faster than the initial one).
Control muscle	m. abductor policis brevis (APB) or m. abductor hallucis (AH)
Pulse type	Rectangular
Pulse duration	0.2 ms
Pulsing frequency	1 Hz
Time delay during simultaneous TVMS+ES: NMES/FES stimulation	0,3 msec (leading stimulus)
Localisation of the stimulation point in relation to TMS stimulation	Contralaterally
Localisation of the stimulation point in relation to the TVMS stimulation	Isolateral
The “condensing” effect (26) of the peripheral stimulus and the subsequent imposed maximal muscle contraction in the patient with CSCI, as well as arbitrary facilitation at full strength, facilitated the conduction of excitation through the pyramidal tracts and, within the course, led to the stable improvements in NF indicators described above, which correlated with the above clinical positive dynamics. In each case, the recorded D-I1 interval remained individually stable, on average within 1–1.5 ms. In addition, it should be noted that when these techniques were combined, the number of TVMS stimuli did not exceed 1000, so the number of electrical stimulation stimuli was also 1000.	
Electroneurostimulation was performed in 2 stages when stimulating the peripheral nerves of the legs. First, we performed dynamogenic training/training of the muscles of the lower and upper extremities (LU) in a serial mode with a rhythm of 1–4 s (with pauses for muscle rest), a frequency of 20–120 Hz, a current of 40–100 mA, then continued in a serial mode with a rhythm of 5–8 s, a frequency of 30–100 Hz, a current of 40–100 mA. The total duration of the session averaged 45 min.	

NMES: neuromuscular electrical stimulation; FES: functional electrical stimulation; TVMS: transvertebral magnetic stimulation.

Analysis of the clinical outcomes observed under both protocol variants revealed comparable therapeutic efficacy; however, a fundamental distinction existed in the criteria guiding protocol selection. The choice between variants was based on individual parameters of central motor conduction and motor evoked potential. Reference values were derived from each patient’s upper limb conduction characteristics. Specifically, in cases where patients exhibited elevated central motor threshold responses, protocol variant “a” was preferred. Conversely, for those with lower thresholds or values consistent with normative literature, variant “b” was selected.

Approximately 28% (*N*_I_ = 43, *n*_I–1_ = 12) of patients in group A moved to a higher level of functioning after 3 courses of high-intensity neurorehabilitation (90 working days). At the end of the first year, ceteris paribus, the number of such cases increased to 38.9% (*n*_I-1 =_ 17). This figure was on average 7–11% higher for cases with low level of spine and spinal cord injuries.

A similarly positive trend was observed in group B patients, with 46.4% (*N*_II_ = 56, *n*_II–1_ = 26) of cases achieving a higher level of functioning over the same period, including an increase to 57.1% (*n*_II-1_ = 32) of cases by the end of the year. The results were even better for the low injury group, with an average of 13% of cases.

Accordingly, 47.3% (*N*_III_ = 38, *n*_III-1_ = 18) of patients with an initial Group C status improved their sensorimotor abilities, with 10 more people joining them by the end of the year.

It is noteworthy that in 73.4% of cases, the same patients showed recovery to almost pre-morbid levels by the end of the second year.

The best results were, of course, achieved by the patients in group D, of whom 64.7% (*N*_IV_ = 17, *n*_IV-1_ = 11) had recovered to level E by the end of the first year of rehabilitation (at the end of the third month; this figure was slightly more than 50%), with minimal neurological symptoms, but not limiting vital activity, according to the survey. The remaining patients in group D (except for 2 patients who did not improve) recovered to level E with minimal neurological symptoms only at the end of the second year of follow-up.

After 3 months of neurorehabilitation, 64 (41%) patients showed improvement in function and neurological status (FRANKEL/ASIA scale), and 100 (65%) showed improvement by the 12-month mark. Patients who received non-invasive neuromodulation using the “end-to-end” protocol 3 times a day for the first 3 months had a significantly better outcome.

Analysis of clinical cases in all study groups, except for group A, which was not included in the main set of results, does not give grounds to consider the proposed approach ineffective, despite the use of identical protocols of non-invasive neuromodulation within the early intensive neurorehabilitation programme. On the contrary, these patients showed positive dynamics, albeit with a significant delay in achieving functional improvement.

At the same time, in 41% of cases in group A (*N*_I_ = 43, *n*_I–2_ = 18), no functional changes were recorded either during the first 9 months after the start of neurorehabilitation or at the end of the first or third year of observation, despite the absence of complete anatomical interruption of the spinal cord at the time of the initial assessment.

## DISCUSSION

The proposed ”end-to-end” neuromodulation protocol for spinal cord function neuromodulation changes the clinical practice of stimulation technologies.

We searched PubMed for the keywords “rTvMS, in spinal cord injury” over the last 10 years (2014–2024) and found only 3 relevant publications. None of them dealt with transvertebral magnetic stimulation in combat-related spinal cord injury (SCI), and the clinical cases described did not cover the acute phase of traumatic injury. The authors note that functional improvement occurs through neuromodulation based on neuroplastic mechanisms. They emphasise the technical simplicity, safety and effectiveness of using an external magnetic field to activate central structures. The process and effects of its use are compared to electrical stimulation of the brain and spinal cord with direct current.

A review of recent studies in the field of SCI treatment indicates the need for further research in the field of non-invasive neuromodulation therapy and neurorehabilitation to improve the quality of life of people with spinal cord injuries.

Our study complements the limited data on neurorehabilitation methods in patients with severe spinal cord injuries. The results are relevant given our country’s sharp increase in such patients. This category is a good clinical model for finding new ways to use technologies, since anything that demonstrates effectiveness in recovering combat spinal cord injuries can be easily transferred to civilian practice, where such injuries are easier to treat and have a better prognosis.

The principle of Gebb’s theory (12, 13), which we used to create a targeted effect on the restoration (improvement) of conductivity in the spinal cord, proved to be the most successful for the set goal.

First formulated by Donald Hebb in 1949, Hebb’s theory remains a fundamental principle of neuroscience, describing the mechanism by which synaptic connections are strengthened through experience. The classic maxim – “neurons that fire together wire together” – generalises that repeated and synchronous activation of presynaptic and postsynaptic neurons leads to long-term potentiation (LTP), the neurophysiological correlate of learning and memory. In neurorehabilitation, Hebb’s theory provides a mechanistic explanation of how repeated, task-oriented stimulation can promote neuroplastic reorganisation, functional recovery and the formation of compensatory pathways. Thus, Hebb’s plasticity underlies the rationale for combining neuromodulation protocols in this study. Fig. 3 shows the application of Hebb’s theory in the proposed neuromodulation protocol. The concept of “end-to-end” stimulation involves the simultaneous delivery of electromagnetic pulses from both ends of the damaged neural pathway to each other. This approach enhances both temporal and spatial coactivation of neural elements, thereby promoting the restoration or enhancement of functional connectivity. In this context, “conductor” refers to anatomically intact but functionally impaired neural components (e.g. axons or neural clusters) whose conductivity may be temporarily suppressed.

Timely bilateral stimulation in the acute post-traumatic phase – before the onset of secondary degeneration mediated by microglial cells – may promote the survival of viable pathways and prevent irreversible conductivity loss.

It is known that combat injuries to the spinal cord are often accompanied by the presence of postoperative soft tissue wounds, which do not heal quickly and require constant medical intervention.

This severely limits the use of classical contact electrical stimulation of the spinal cord, especially in the acute period, since using contact electrodes in these conditions is contraindicated. In addition, transcutaneous delivery of direct current through electrodes to physiological targets has significant physical disadvantages, such as dispersion, leakage, skin resistance and skin-electrode impedance.

The clinical advantage of magnetic stimulation in patients with combat spinal cord injuries is further enhanced by its non-invasive ability to affect the affected area without the need to remove bandages or come into contact with damaged skin. In addition, magnetic stimulation does not depend on skin impedance and provides deep penetration of the stimulus, making it technically superior to traditional electrical stimulation (27).

Studies have shown that magnetic stimulation causes less discomfort and pain in patients, especially in the cervical region, than electrical stimulation (28).

The simultaneous combination of transvertebral magnetic stimulation with electrical stimulation of peripheral nerves allows Gebb’s theory to be put into practice, enhancing the neuromodulatory effect through synchronised activation of neural circuits. In combination with robot-assisted kinesitherapy, this combined strategy demonstrates clear advantages in the restoration of motor functions, especially in the acute post-traumatic phase.

Unlike the isolated use of magnetic stimulation (29) or other non-invasive neuromodulation protocols, including the isolated use of electrical stimulation, this approach provides better spatial and temporal stimulation coordination, offering a clear clinical advantage in such cases.

Despite the promise of rTvMS as a non-invasive neuromodulation technique, certain limitations must be acknowledged. First, the mechanisms by which rTvMS affects the spinal cord are not yet fully understood, making it challenging to predict therapeutic outcomes and optimise stimulation parameters accurately. Second, the effectiveness of rTvMS may be limited by individual anatomical features, such as subcutaneous fat thickness and vertebral position, which affect the depth of magnetic field penetration. In addition, the lack of standardised protocols and the limited number of large-scale clinical studies hinder the widespread implementation of rTvMS in clinical practice (30).

The proposed “comprehensive’ neuromodulation protocol represents a paradigm shift in the clinical application of spinal cord stimulation technologies. It is worth noting that functional and neurological improvements, as assessed by the FRANKEL/ASIA scale, were found in 64 patients (41%) after 3 months and in 100 patients (64.9%) after 12 months of follow-up, predominantly in those who received the highest number of neuromodulation sessions.

These results highlight the potential of synchronised bilateral stimulation to improve neuroplastic recovery in acute SCI.

At the same time, the absence of functional improvement in 41% of cases in Group A (*N*_I_ = 43, *n*_I-2_ = 18) – despite the application of neuromodulation technologies and the lack of neuroimaging evidence of complete anatomical transection of the spinal cord – indicates that gunshot-related spinal cord injuries continue to represent the most severe form of spinal trauma, with a high likelihood of an unfavorable prognosis.

In summary, given the increasing number of patients with severe combat-related spinal cord injuries in Ukraine and worldwide, our work complements the limited database and justifies the need for further research in this field. The knowledge gained can be easily adapted to the needs of civilian medicine, where similar injuries have a better prognosis and are less complex to treat. Further in-depth study of non-invasive neuromodulation, particularly rTvMS, is critical and already planned by our team..

### Clinical implications/future directions

This study shows how effective high-intensity neurorehabilitation can be for casualties with high-energy spinal cord injuries from modern combat (mine-explosive) in the acute phase, considering the injury’s severity, extent and history. We have combined repetitive transvertebral magnetic stimulation and simultaneous synchronised transcutaneous electrical stimulation of peripheral nerves in NMES and FES modes with innovative hardware kinesitherapy, including robotic kinesitherapy with biofeedback, to improve sensorimotor functions and activities. Further studies are needed to clarify the neurorehabilitation process with TMS aspects in subsequent studies.

### Evidence before this study

We searched PubMed for studies published in the last 10 years using the search terms «transvertebral magnetic stimulation for spinal cord injury» without language restrictions and found only 3 articles. None of them mentioned transvertebral magnetic stimulation of the spinal cord. It is not indicated that the cases analysed were in the acute period of injury. However, the main thing was that these publications do not consider gunshot (mine-blast) spinal cord injuries, the course of which is proven to be different from civilian trauma. These papers describe various methods of direct current stimulation of the spinal cord, including robotic kinesitherapy, in combination with direct current stimulation of the motor cortex. Based on an understanding of the morphological, functional and cellular processes of “neuroplasticity,” the authors attribute the improvements in the functioning of the victims to neuromodulation. In addition, they point out the safety, relative technical simplicity and effectiveness of using an external alternating (high frequency) magnetic field to modulate brain activity in various diseases, which contributed to the expansion of indications for using magnetic fields in spinal cord injuries. At the same time, the process and effects of its use are compared with electrical stimulation of the brain and spinal cord with direct current.

Next, we changed the search parameters by adding the words “electrical direct current stimulation of spinal cord injury” and naturally found many more publications during the same search period – 116. These papers highlight various positive aspects of the use of electrical stimulation of the spinal cord with direct current, both during and after surgery, which relate to either the improvement of certain neurophysiological parameters of sensorimotor conduction or, in general, indicate an increase in motor capabilities in patients at different periods of injury or improvement of sensory and autonomic functions or sacral functions (S4–S5 segments of the spinal cord) or reduction of pain or spasticity. However, the same as in the previous search for a focused study of cases of influence on the restoration of functions in the acute period of gunshot SCI is not noted.

The low risks of complications from using direct electric current, relative technical simplicity and even availability for home use are also described. Adding the word “non-invasive” reduced the number of published studies to 20, but we did not find any new information beyond that described above.

Finally, we entered the words “non-invasive electrical stimulation of peripheral nerves for spinal cord injury” in the search and received 21 publications. The authors describe in detail the combination of electrical stimulation of peripheral nerves with various physical exercises – electrically induced physical activity, or electrically induced neuromuscular activity (muscle contractions), often called “neuromuscular electrical stimulation (NMES) and functional electrical stimulation (FES).” This combination, in their opinion, helps to improve the mobility and health of people with SCI by strengthening muscles or increasing functional activity of patients, reducing spasticity, reduces the risk of cardiometabolic conditions associated with impaired vital activity [Leisure time physical activity (LTPA) and helps in predicting hand deformities in people with tetraplegia (grasping movements]. We did not find any reports of cases of electrical stimulation of peripheral nerves in combat gunshot spinal cord injuries in combination with magnetic transvertebral spinal cord stimulation.

The evidence supporting the effectiveness of using magnetic fields and direct current to stimulate peripheral nerves in the acute period of injury in combination with other neurorehabilitation methods to improve sensorimotor and other vital functions, which enhance the quality of life and prognosis, remains limited. This is not to say, however, that we could not find any reports on CSCI and the use of these methods in its treatment.

Further convincing evidence is needed to confirm the effectiveness of simultaneous (synchronised) use of such potentially attractive alternative methods of non-invasive neuromodulation as transvertebral rhythmic magnetic stimulation of the spinal cord and transcutaneous electrical stimulation of peripheral nerves in NMES/FES modes, which are directly related to the topographic level of injury in the acute period when the window of opportunity is most comprehensive.

### Added value of this study

It is also important to note that almost every paper we found during our evidence collection, in its ‘Objective’ section, directly or indirectly points to the severe (catastrophic) long-term suffering of patients with spinal cord injuries, poor quality of life, shortened life expectancy and even premature death, despite the development of medicine in recent years. It is emphasised that the only way to improve their condition is neurorehabilitation, especially early neurophysiological rehabilitation, because traditional physical rehabilitation, due to the specifics of SCI, is usually ineffective.

The results of these few studies do not idealise the situation with SCI care but clearly emphasise the need for further search and study of the impact of new, alternative strategies for using non-invasive neuromodulation therapy in combination with neurorehabilitation methods to improve the quality of life in patients with SCI. In this sense, our study and its conclusions, based on the example of combat gunshot SCI in the acute period of trauma, which, all things being equal, has a more pronounced clinical manifestation, worse course and prognosis, are particularly informative and indicative for further studying the effects of non-invasive neuromodulation of spinal cord functions using advanced electromagnetic technologies and searching for new approaches, which is the principle of synchronous end-to-end stimulation proposed by us. Even the realisation that the presence of numerous purulent mine-blast soft tissue wounds that do not heal for a long time and require constant medical intervention in the area of interest for spinal cord stimulation is already a contraindication for the use of contact electrodes (stickers), not to mention the significant and challenging to overcome physical disadvantages of the transcutaneous passage of direct electric current through electrodes to physiological targets (such as scattering effect, leakage, skin resistance and skin-electrode impedance) in spinal patients, allow us to confidently use rhythmic transvertebral magnetic stimulation in such cases and study the facts of its effect.

### Implications of all the available evidence

There are no or minimal studies dedicated to the search for effective tactics of early intensive neurorehabilitation of victims with modern combat high-energy gunshot (mine-blast) SCI in the acute period of injury, taking into account the specifics of this type of injury (serious condition, widespread wounds, damage to other areas, numerous repeated operations, etc.). Rhythmic transvertebral magnetic stimulation and transcutaneous electrical stimulation of peripheral nerves in NMES and FES modes with innovative methods of hardware kinesitherapy, including robotic kinesitherapy with biofeedback to improve, first of all, sensorimotor functions and vital activity, against the background of increasing military conflicts and increasing militarisation of countries, create all the necessary grounds and evidence to begin to clarify these aspects in this study.
